# Conditioned pain modulation is associated with heightened connectivity between the periaqueductal grey and cortical regions

**DOI:** 10.1097/PR9.0000000000000999

**Published:** 2022-05-07

**Authors:** Richard Harrison, Wiebke Gandhi, Carien M. van Reekum, Tim V. Salomons

**Affiliations:** a University of Reading, School of Psychology and Clinical Language Sciences, Reading, United Kingdom; b Department of Psychology, Queen's University, Kingston, ON, Canada

**Keywords:** Conditioned pain modulation, fMRI, Resting state, Individual differences, Periaqueductal grey

## Abstract

**Introduction:**

Conditioned pain modulation (CPM) is a psychophysical assessment used to estimate the efficiency of an individual's endogenous modulatory mechanisms. Conditioned pain modulation has been used as a predictive assessment for the development of chronic pain and responses to pain interventions. Although much is known about the spinal cord mechanisms associated with descending pain modulation, less is known about the contribution of supraspinal and especially cortical regions.

**Objectives:**

We aimed to explore how whole-brain connectivity of a core modulatory region, the periaqueductal grey (PAG), is associated with conditioned pain modulation, and endogenous pain modulation more broadly.

**Methods:**

We measured CPM and resting-state connectivity of 35 healthy volunteers, absent of chronic pain diagnoses. As a region of interest, we targeted the PAG, which is directly involved in endogenous modulation of input to the spinal cord and is a key node within the descending pain modulation network.

**Results:**

We found that CPM was associated with heightened connectivity between the PAG and key regions associated with pain processing and inhibition, such as the primary and secondary somatosensory cortices, as well as the motor, premotor, and dorsolateral prefrontal cortices. These findings are consistent with connectivity findings in other resting-state and event-related fMRI studies.

**Conclusion:**

These findings indicate that individuals who are efficient modulators have greater functional connectivity between the PAG and regions involved in processing pain. The heightened connectivity of these regions may contribute to the beneficial outcomes in clinical pain management, as quantified by CPM. These results may function as brain-based biomarkers for vulnerability or resilience to pain.

## Abstract 2

Resting-state functional connectivity between the periacqueductal gray and cortical regions involved in sensory, motor, and cognitive processing is associated with conditioned pain modulation.

## 1. Introduction

Original Cartesian models of pain posited a direct relationship between noxious input and perceived pain, whereas subsequent research has shown that pain is not just a reflection of injury but can be regulated endogenously.^1,26,30,33^ Given this, the traditional medical approach of matching injury to symptomology (and vice versa) seems increasingly inappropriate within pain diagnostics. The variable link between pain and pathology necessitates study into why some patients with subdiagnostic pathology are vulnerable to pain, whereas others seem more resilient.

One approach to understanding this vulnerability is to assess the efficiency of an individual's endogenous modulatory mechanisms. Conditioned pain modulation (CPM)^43^ is one such assessment that was developed to test diffuse noxious inhibitory control (DNIC), whereby neural response to one noxious input to the dorsal horn of the spinal cord is inhibited by noxious input from another somatic location.^14,24^ Although much is known about the spinal cord mechanisms of DNIC, less is known about the contribution of supraspinal and especially cortical regions.

**Figure 1.**
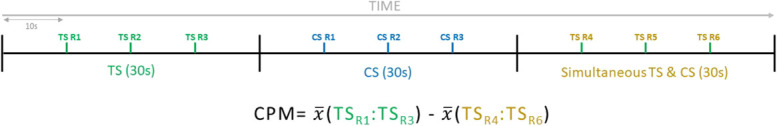
Method for calculating conditioned pain modulation (CPM) scores using a comparison of ratings with the test stimulus (TS) administered in isolation and during concurrent stimulation through a conditioning stimulus (CS).

Conditioned Pain Modulation involves the simultaneous presentation of 2 stimuli coalescing to form a modulatory effect, reflecting the extent to which pain ratings of a test stimulus are reduced when combined with a second conditioned stimulus (Fig. 1). Task-based functional magnetic resonance imaging (fMRI) studies have shown that this decrease in ratings is paralleled with decreased BOLD responses in key pain processing regions, such as the thalamus, somatosensory cortex, and dorsolateral prefrontal cortex (DLPFC)^3,31,38,39^ but with an enhanced BOLD response in the perigenual anterior cingulate cortex (ACC) and periaqueductal grey (PAG).^24,38^ The PAG is directly involved in endogenous modulation of input to the spinal cord^4,13,28,41^ and is a key node within the descending pain modulation network.^25,27,29^ Of importance, the PAG is bidirectionally connected to cortical regions involved in processing pain, including the DLPFC, anterior cingulate and insula cortices, thalamus and precuneus.^10,23,41^

Despite the need to better understand the brain's contribution to CPM effects, doing so using task-based fMRI is difficult because dissociating the neural responses to the 2 experimental stimuli (as well as nonadditive modulatory processes) is not straightforward. An alternative imaging approach is resting-state fMRI (rs-fMRI), which allows for the evaluation of interindividual variation in pain modulation capability at rest. This approach provides insight into brain connectivity differences that might be associated with interindividual variation in CPM.^34,37^ Harper et al. identified lower grey matter density in the PAG in patients with fibromyalgia compared with that in healthy controls.^17^ They then assessed rs-fMRI connectivity of the PAG across groups and found that CPM was associated with higher connectivity with regions associated with pain inhibition, including the pregenual ACC and mid-insula, suggesting this functional integration facilitates pain modulation.

Harper et al.^17^ examined PAG connectivity in a combined group of patients and controls. To further elucidate individual differences in pain modulatory processes, this article used the same seed-based approach, but in a sample of healthy controls. We predict that CPM will be associated with a higher connectivity between PAG and regions associated with pain processing and inhibition.

## 2. Methods

### 2.1. Participants

Forty healthy individuals were recruited from the University of Reading and were excluded if they experienced active or historical chronic pain disorder diagnoses, current substance abuse, uncorrected visual defects, or left-hand dominance. Five participants were excluded (3 could not tolerate the conditioning stimulus within the CPM paradigm and 2 experienced excessive motion artifacts during resting state [>2.5 mm]), leaving a final sample of 35 participants (14 female participants; M^age^ = 22.8 years, SD = 5.53). All participants provided informed consent before the study and received course credits in payment for their participation. The study was approved by the University of Reading's Research Ethics Committee.

## 3. Materials

Noxious heat stimulation was administered through a MEDOC Pathway system (Medoc Medical Systems, Ramat Yishai, Israel) with a 30 × 30 Peltier thermode applied to the lower right calf, which was placed into a custom-made wooden leg rest. The test stimulus was calibrated to represent a 6/10 pain intensity rating for each participant (see further for calibration method). The conditioning stimulus was elicited by a Julabo TW20 water bath set at 46.5°C.

### 3.1. Design

This experiment was completed as part of a larger 4-session study. The first session was a psychophysical assessment. A separate neuroimaging session was completed no more than 7 days after this initial session. Lastly, 2 sessions consisting of cognitive and emotional pain modulation tasks (not described here) were run. Within the neuroimaging session, an rs-fMRI scan was run immediately after an initial localiser. In addition, 4 runs of an event-related functional task (not described here) were completed, with a T1-weighted structural scan acquired after 2 of the 4 runs. Task findings have been reported elsewhere.^11,18^

### 3.2. Procedure

#### 3.2.1. Pain threshold assessment

To calibrate each participant's pain threshold, we used a combination of 2 separate techniques. Both methods used the same visual analogue scale.^32^ The minimum rating of 0 was described as no pain at all, whereas the highest anchor of 10 was anchored with most intense pain imaginable, and the scale was displayed to the participant using a piece of laminated A4 paper. For a full description of the thresholding methods, see Harrison et al., 2018 (p.3).^18^

#### 3.2.2. Temperature calibration

To calibrate the CPM test stimulus, the next stage of the assessment consisted of a ranged-temperature stimulus response curve. A total of 9 ranges were available, using threshold as the median value in a range of temperatures separated by 0.75°C with 4 temperatures above threshold and 3 below (ie, mid-2.25°C, mid-1.50°C, mid-0.75°C, mid, mid+0.75°C, mid+1.50°C, mid+2.25°C, mid+3°C). Each temperature within the range was presented 3 times, equalling a total of 24 stimuli. Participants were told to rate the intensity of each stimulus using the same 0 to 10 visual analogue scale as before. The stimuli were presented for 8 seconds, after which a rating was collected. Each temperature was separated by an interstimulus duration of 20 seconds to limit the influence of habituation or sensitisation on the proceeding stimuli.

Once all ratings had been recorded, the pairings of temperature and pain ratings were entered into an online linear regression calculator. The model generated from the stimulus response curve data allowed us to interpolate which temperature best matched each participant's indicated 6 of 10, based on their pain ratings to all temperatures within their range.

#### 3.2.3. Conditioned pain modulation

Our CPM paradigm was based on previously published material.^15,43,45^ The interpolated temperature for the participant's 6/10 was designated as the test stimulus. The first trial consisted of the test stimulus in isolation for 30 seconds, with a total of 3 pain intensity ratings provided at 10 second-intervals. The second trial started with the participant submerging their left hand and wrist into the water bath where they provided 3 ratings of the pain intensity of the conditioning stimulus at 10 second-intervals. Once these 30 seconds had elapsed, the participant continued to keep their hand in the water bath and provided 3 more ratings of pain intensity of the test stimulus at 10 second-intervals. Finally, the participant provided a final conditioning stimulus pain rating once the test stimulus has ended.

#### 3.2.4. Functional magnetic resonance imaging acquisition

Brain images were acquired using a 3T Siemens (Siemens, Erlangen, Germany) TRIO magnetic resonance imaging (MRI) scanner with a 32-channel head coil. For the 10-minute resting-state scan, participants were instructed to keep their eyes closed. The protocol consisted of 30 interleaved 3.5-mm sagittal T2*-weighted gradient echo echo-planar imaging slices (TE = 28 ms, TR = 2000 ms, flip angle = 90°, 1-mm interslice gap; 128 × 128 matrix, field of view = 240 mm). Consequently, 300 volumes were acquired and then prepared as 4D NIFTI images. Structural images were then acquired within an 8-minute T1-weighted inversion recovery fast gradient echo-high resolution structural scan (176 volumes, TE = 2.9 ms, TR = 2000 ms, flip angle = 90°, voxel size = 1 × 1 × 1; 256 × 256 matrix, field of view = 250 mm).

### 3.3. Functional magnetic resonance imaging data analysis

#### 3.3.1. Region-of-interest selection

**Figure 2.**
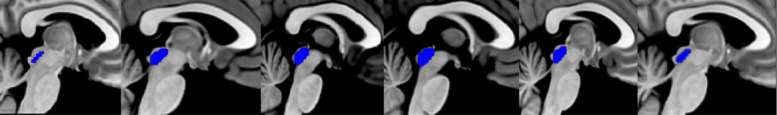
Periaqueductal grey region-of-interest (blue) across sagittal view (left to right; x = −5, −3, −1, 1, 3, 5).

Owing to the risks of a functionally derived PAG seed experiencing activation leak into the cerebral aqueduct, for the purpose of preparing this region-of-interest (ROI), a PAG seed encompassing 133 voxels was hand-drawn onto the MNI template in the grey matter surrounding the cerebral aqueduct within the tegmentum of the midbrain. This ROI coalesced with the area previously described and reported for the human PAG (Fig. 2).^9^ A control ROI was also included to increase confidence in the specificity of the results to the experimental ROI. For this purpose, a region with no related function to the process of CPM was required. As such, the temporal pole was selected because it is primarily associated with functions such as face recognition, autobiographic memory, and word–object naming.^19^ The bilateral mask was created using the Harvard-Oxford Cortical Structural Atlas and binarised with a 50% probabilistic threshold.

#### 3.3.2. Preprocessing

Analysis was performed using the FMRIB software library package (FSL version 6.00; www.fmrib.ox.ac.uk/fsl).^21^ The Brain Extraction Tool^36^ was used for skull stripping. The first 5 volumes were removed to allow for signal equilibration effects. An interleaved slice-timing correction was applied. Data were smoothed with a 5-mm full-width half-maximum Gaussian spatial smoothing kernel. MCFLIRT was used for motion correction,^20^ and data were visually inspected for motion artifacts and to confirm registration accuracy.

In line with recommendations for correcting for physiological and movement-related noise, especially regarding resting-state data, the component-based noise correction method (COMPCOR) was performed, which regresses out non-neuronal noise signals through white matter and CSF segmentation, which are unlikely to contain signal modulated by neuronal activity. COMPCORR has been shown to be amongst the best practice for controlling for physiological noise in fMRI data and performs at levels similar to retrospective image correction through physiological recordings.^2^

Grey matter was segmented from white matter (WM) and cerebrospinal fluid (CSF) using FSL FAST module^47^ to regress out signal associated with WM and CSF as nuisance variables. To minimize overlapping signal from grey matter before time series extraction, WM and CSF maps were thresholded at 0.99. Time series of WM and CSF were entered into a general linear model along with motion parameters. Residuals from this nuisance analysis were normalised and bandpass filtered (0.1/0.01 Hz) to reduce the influence of low-frequency drift (inclusive of scanner drift) and high-frequency interference such as cardiac or respiratory confounds.

### 3.4. Resting-state analysis

The mean time series of all voxels within our PAG seed were extracted and included as a regressor in a whole-brain functional connectivity analysis. Contrast images were then entered into second higher-level analyses with participant's demeaned CPM scores entered as a regressor. Because the MRI session occurred on a different day to the psychophysical pain assessment, to control for a potential temporal confound, the demeaned number of days between assessment and MRI scan were added as a covariate within the same general linear model. This analysis sought regions where connectivity with the PAG was significantly associated with CPM. All fMRI analyses were corrected for multiple comparisons using nonparametric permutation inference testing (*P* < 0.05) with threshold-free cluster enhancement, using FSL randomize module.^40^ For the purposes of graphical presentation of our results, parameter estimates from regions where functional connectivity with the PAG was significantly correlated with CPM were extracted using FEATQuery.

## 4. Results

### 4.1. Conditioned pain modulation

Participants rated the pain intensity of the thermode to be 5.93 (SD = 1.49). The mean stimulus temperature across the sample to elicit a 6/10 rating was 45.7°C (SD = 1.56). When combined with a conditioning stimulus, the rating for the thermode decreased to 4.27 (SD = 2.0), indicating a significant reduction of 1.65 (SD = 1.38) in pain intensity (t(33) = 6.97, *P* < 0.001). Each individual's difference score was used as their CPM score with higher CPM scores representing more effective pain modulation (**Fig. 1**).

### 4.2. Resting-state connectivity & conditioned pain modulation

**Figure 3.**
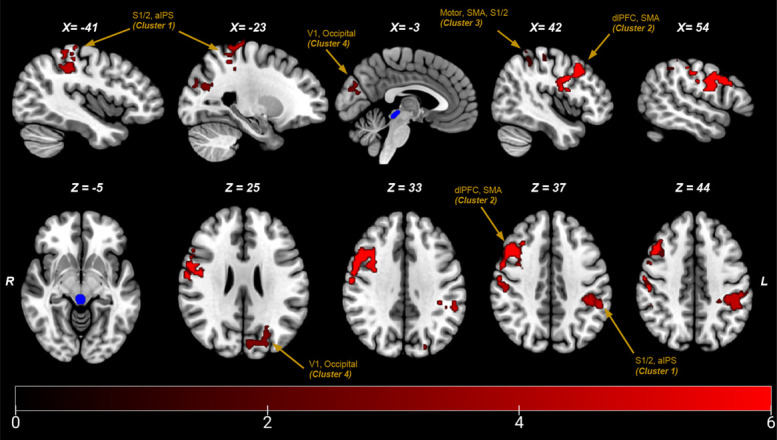
Isolated clusters (numbered in relation to clusters in table) in radiological view in MNI space positively correlated with CPM (thresholded at *P* < 0.05, Z = 2.3). PAG seed displayed in blue. aIPS, anterior intraparietal sulcus; CPM, conditioned pain modulation; dlPFC, dorsolateral prefrontal cortex; PAG, periaqueductal grey; S1/2, somatosensory cortices; SMA, supplementary motor area.

In total, there were 4 clusters of activation where PAG connectivity was positively correlated with participants' CPM scores (Fig. 3 and Table 1), whereas no negative correlations survived thresholding. These included the bilateral primary and secondary somatosensory clusters (clusters 1 and 3), as well as the right motor and premotor cortices (cluster 3). Cluster 2 consisted predominantly of the dorsolateral prefrontal cortex but also extended posteriorly into the motor, supplementary motor, and somatosensory cortices.

**Table 1:** Statistical peaks in MNI space associated with positive connectivity to the periaqueductal grey and conditioned pain modulation scores, while controlling for time between pain assessment and magnetic resonance imaging.

Anatomical brain region	Brodmann areas	MNI coordinates			Max Z-stat
		X	Y	Z	
1. Left somatosensory cortex 1 (S1) & 2 (S2), inferior parietal and anterior intraparietal sulcus (aIPS)	BA1, BA2, BA3b	−44	−34	44	4.67
2. Right dorsolateral prefrontal cortex (DLPFC), primary and premotor cortices	BA44, BA46	58	0	26	5.4
3. Right primary motor and premotor cortices, S1 & S2	BA3a,BA3b,BA4a, BA4p, BA6	36	−38	70	4.58
4. Left occipital cortex, V1 and superior parietal	BA17 & BA18	−22	−82	28	3.87

Coordinates provided at site of maximum Z-stat.

**Figure 4.**
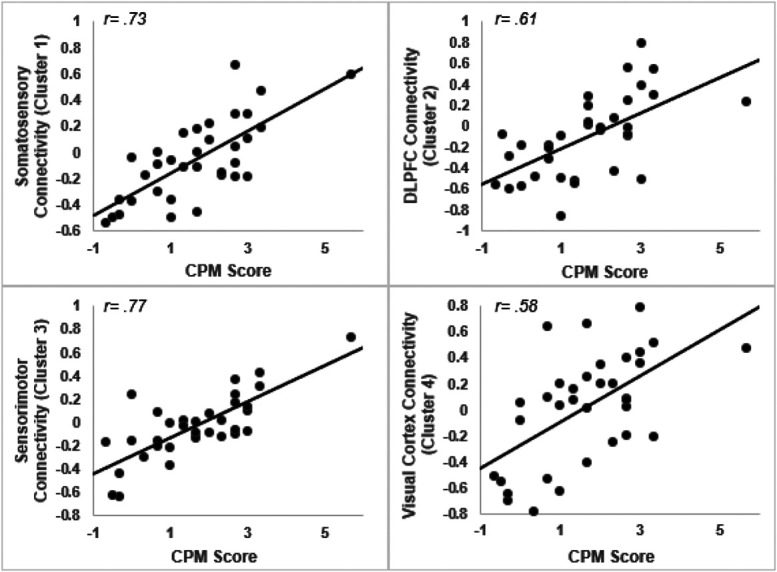
Connectivity of the PAG and all extracted clusters correlated with CPM scores, as listed in Table 1, with higher CPM scores representing more effective pain modulation. CPM, conditioned pain modulation; PAG, periaqueductal grey.

The association between CPM and all 4 extracted clusters is plotted in Figure 4. Owing to the presence of a single outlier that scored highly on CPM, all correlations were retested with the extreme value excluded, and all correlations remained significant. Regarding the control ROI, no significant clusters were identified that were associated with CPM, as in the experimental ROI analysis.

## 5. Discussion

Conditioned pain modulation is a psychophysical assessment of the efficiency of endogenous modulatory pathways in humans.^44^ This study investigated whether intrinsic resting-state connectivity of the PAG (a key pain modulatory region) was associated with CPM in healthy controls. In line with our hypotheses, we found that CPM was associated with heightened integration of the PAG and somatosensory cortices, premotor and motor cortices, and the DLPFC, all regions associated with the processing or modulation of pain.^4,5,7,12,27,29^ These findings partially corroborate those of Harper et al., who identified a similar functional integration of regions associated with pain processing and modulation.

Our findings suggest that individuals who are efficient modulators have greater functional connectivity between the PAG and regions involved in processing pain. The PAG is a key region in the modulation of pain,^29^ and the cortex and PAG have a bidirectional relationship that can affect the modulatory process itself.^6,41^ The PAG is functionally connected with the dorsolateral prefrontal, motor, and somatosensory cortices at rest,^10^ and our results indicate that when this functional connectivity is stronger, people are able to more effectively modulate their pain. This influence is likely to be bidirectional because the PAG not only modulates the ascending signal at the level of the spinal cord but also is involved in preparing the body to deal with noxious stimuli.^35^ This may also involve communication with sensory and motor areas, better enabling the individual to attend to the stimulus itself and prepare for adequate nocifensive action.

Similar to a previous study by Harper et al.,^17^ we found connectivity of the PAG to be associated with individual differences in CPM. There were differences, however, between the connected regions found to correlate. These discrepancies may be due to methodological differences (eg, mechanical vs thermal CPM paradigm, functionally vs anatomically derived PAG seed map) but are more likely due to differences in the study populations, as the findings by Harper et al. were derived by combining healthy controls with fibromyalgia patients, whereas we examined only individuals without chronic pain. This is especially pertinent due to the identification of morphological differences of the PAG between the 2 groups within the VBM-based seed formulation process. Further research is needed to elucidate how living with chronic pain (and, more specifically, different types of chronic pain) may change the capacity for pain modulation at the neural and behavioural levels.

Promisingly, our findings complement the limited evidence on CPM mechanisms derived from studies using imaging during CPM. The challenges of imaging CPM using an event-related design are substantial. The equipment used when assessing CPM can be restrictive when applying to an MRI environment (eg, water baths, thermal stimulators, and metallic algometers), and the combinative stimulation of test and conditioning stimulus can make isolating the pure influence of modulation difficult. However, within the available literature, it has been reported that during concurrent conditioning and test stimuli presentation, the dorsolateral prefrontal, premotor, and primary and secondary somatosensory cortices show reductions in BOLD response, in parallel to reductions in pain intensity elicited by the CPM effect.^3,31,38,39,46^

Of particular relevance to our study is an event-related study by Piche et al., which also investigated functional connectivity.^31^ Results of the study by Piche et al. complement our findings, identifying functional coactivation between a PAG seed and the somatosensory, premotor, motor, and prefrontal cortices. The variability of CPM methodological design remains an ongoing issue for the interpretation, comparison, and reliability of findings in the field^22^ and standardisation of the design would likely improve the robustness of our conclusions.^42^ Nevertheless, correspondence between our findings and these event-related designs suggests the possibility that the patterns of resting-state functional connectivity found to be associated with individual differences in pain modulation in our study may be directly involved in CPM.

This study provides insight into CPM as a measure of the efficiency of pain modulation circuitry. CPM has been used as a predictive assessment for postsurgical outcomes, analgesic efficiency, and the risk of developing neuropathic pain,^8,16,43,45^ indicating clinical applicability. This suggests that our results, alongside other fMRI studies of CPM cited here, may function as brain-based biomarkers for vulnerability or resilience to pain. Future research should therefore investigate whether resting-state connectivity between PAG and cortical regions involved in the processing and modulation of nociceptive input is useful in predicting clinical outcomes.

## Disclosures

R. Harrison was funded by a joint UK National Health Service-University of Reading CASE studentship and received postdoctoral funding from a New Investigator Research Grant from the Medical Research Council of the UK to TVS (MR/R005656/1). W. Gandhi is funded by a Leverhulme Early Career Fellowship. T.V. Salomons was funded by a Marie Curie International Incoming Fellowship, a British Academy/Leverhulme Small Grant, and a New Investigator Research Grant from the Medical Research Council of the UK to TVS (MR/R005656/1). The remaining author has no conflicts of interest to declare.
